# Genetics of cognitive performance, education and learning: from research to policy?

**DOI:** 10.1038/s41539-022-00124-z

**Published:** 2022-05-04

**Authors:** Peter M. Visscher

**Affiliations:** Institute for Molecular Bioscience, The University of Queensland, St Lucia, QLD 4072 Australia

**Keywords:** Neuroscience

Genetic variation for ability and learning is ubiquitous in animals, including in humans for physical, cognitive and social abilities. Empirical evidence supporting the thesis that individual differences in humans for cognitive ability and socio-economic outcomes are in part due to genetic factors is overwhelming^1^. It is nicely summarised in Paige Harden’s book “The Genetic Lottery: Why DNA Matters for Social Equality”^2^. Genetic variation underlying human traits is the result of two genetic lotteries. Firstly, it is the lottery of who your biological parents are and secondly it is the lottery of which genetic variants you have inherited from your parents. The first explains a proportion of the differences between families whereas the latter explains a proportion of the differences between brothers and sisters. Nature is not fair, hence the title of Harden’s book.

An example of empirical data on genetics and social outcomes comes from the latest genome-wide association study (GWAS) of educational attainment^3^. A GWAS is an experimental design where millions of DNA variants in the genome are tested for association with traits measured on a (large) sample of individuals in the population. The latest GWAS on educational attainment is from a sample of more than 3 million (!) individuals. The authors (disclosure: I am one of them) report about 3000 DNA variants that reach stringent statistical significance for association. More importantly, a “polygenic score” (PGS) derived from the estimated effects of the DNA variants in the study explains about 15% of individual differences in out-of-sample prediction, among people who are genetically of European descent. This means that the correlation between outcome (the number of years of schooling or attaining college completion) and the PGS is approximately 0.4, using only a DNA sample as information. Another way of looking at these results is to consider the effect size: if the standard deviation (SD) of the number of years of education is 4 years, then the PGS has a SD of 0.4*4 = 1.6 years, which is much larger than the effects on educational attainment estimated from policy changes or randomised intervention studies^2^. Indeed, the difference in prevalence of college completion between individuals in the top decile versus bottom decile of the distribution of the PGS was 50% (60% versus 10%)^3^, which is huge.

Therefore, genes matter when it comes to educational performance and social outcomes (as they do for how tall you are and your risk of many diseases). There are multiple ways of dealing with this “inconvenient truth”, from ignorance or denial to embracing the knowledge and use it in research with the ultimate aim to inform better policy^1^. Two recent papers in this journal fall into this space.

## Tracking in education and inequality of opportunity

Many countries track (stream) children into secondary education based upon their performance at primary school. For policy makers, relevant questions are whether tracking is beneficial and fair and at what age kids should be tracked. Knigge and colleagues use a genetically informed experimental design to investigate whether delayed tracking (streaming) might reduce inequality of opportunity, using a very large number of identical and non-identical twin pairs from the Netherlands^4^. In 1974, when I was in my last year of primary school (aged 12) in the Netherlands, I and other children took a national exam which was used to track students into secondary education. This test is still used today and Knigge *et al*. use data from this test on thousands of twins, some of whom were tracked at a later age, and their attained secondary school level, to quantify the effect of delayed tracking. The “genetics” in this study comes from the twin design: identical twins share all their genetic variants they inherited from their parents whereas non-identical twins only share 50% (on average), and researchers use this difference to partition observed trait variation into genetic, shared environmental and person-specific unique environmental components. Their main result, using state-of-the-art statistical modelling, was that the effect of shared family environment was substantially reduced among twins whose tracking was delayed until a later age. The results are consistent with the hypothesis that delayed streaming improves equality of opportunity by reducing the effect of prior family environmental influence. However, this conclusion is indirect and the design did not lend itself to a direct estimate of the causal effect of delayed tracking on outcomes later in life. The study would have been more powerful if it had used PGS for ability (which the authors measured using the age 12 exam) and PGS for educational attainment. More research is needed to estimate a direct causal effect of the time of tracking on educational outcomes, but this study provides a useful proof-of-principle that links genetics with education policy.

## Genetic effects on learning

The “genetics” in the second study by Youn et al.^5^ comes from using PGS in a novel experimental setting. Here, the authors (the aforementioned Paige Harden is one of them) attempt to quantify and test the effect of a PGS for educational attainment on learning trajectory. The idea is to separate the effect of genetics on cognitive performance (i.e., performance at baseline) from its effect of learning. The authors used data from the COGITO study, in which adults aged 20-80 were measured for memory and perceptual speed at baseline, and repeatedly thereafter using individualised difficulty levels across 100 days, concluding with a post-test assessment^6^. In the paper from Youn et al.^5^ the authors used 9 cognitive tests in a sample of 131 adults from whom they also had a DNA sample and therefore could measure their PGS for educational attainment. The main novelty of this study is in the design because it was too small to draw any meaningful conclusions from the empirical data presented, as recognised by the authors. A sample size of the order of thousands might be required to quantify genetic effects on baseline performance and subsequent learning. Nevertheless, with the power of PGS increasing over time (the latest PGS from Okbay and colleagues^3^ is more powerful than that used by Youn et al.), and larger experimental sample sizes, this kind of longitudinal experiment could be used to dissect the genetics of learning and perhaps identify biologically distinct patterns of learning.

## Embedding genomics into the social sciences and neuroscience

These two new studies are just a snapshot of how researchers can use genetics to address important questions about cognitive performance, learning and social outcomes (including educational attainment). In my opinion, human genomics, in particular the use of polygenic scores, should become fully embedded into social sciences research, including economics, sociology and psychology. If a substantial proportion of the differences between people in important outcomes in life is due to their DNA, then why not utilise this information to design better experiments and to inform policy? In research settings, the use of human genomics data would allow for more powerful experimental designs by accounting for a proportion of variation among samples in the experiment, to detect biases and confounders (e.g., due to genetic ancestry differences between treatment and control groups) and to move from associations to causation. The same principles also apply to studies in neuroscience that use human subjects. Variation in any trait we could measure, including brain imaging data, is partly genetic, and exploiting this fact will lead to a better understanding of the causes and consequences of human behaviour and its underlying biology.

Could genetics inform policy that enhances learning and educational outcomes? Harden describes a number of examples in her book^2^. An extreme example of such policy would be to track individuals into secondary or higher education using, among other sources of information, their genetic predictor for cognitive ability and other traits. Such a “personalised education”^7^ approach is clearly not endorsed widely^2^. However, existing tracking methods that were used when I grew up in the Netherlands (and used in the Knigge et al. study^4^) and are employed in many other countries around the world are highly selective on cognitive performance (ability) and therefore indirectly already selective on genetic factors. Measuring these factors directly through a PGS would allow a fairer comparison across individuals that is not influenced by their socio-economic background.

## Data Availability

There are no data attached to this manuscript.

## References

[1] Harden KP, Koellinger PD (2020). Using genetics for social science. Nat. Hum. Behav..

[2] Harden, K. P. *The Genetic Lottery: Why DNA Matters for Social Equality* (Princeton University Press, 2021).

[3] Okbay, A. Polygenic prediction of educational attainment within and between families from genome-wide association analyses in 3 million individuals. *Nat. Genet.*10.1038/s41588-022-01016-z (2022). 10.1038/s41588-022-01016-z PMC9005349 35361970

[4] Knigge, A. et al. Delayed tracking and inequality of opportunity: Gene-environmental interactions in educational attainment. *NPJ Sci. Learn.*10.1038/s41539-022-00122-1 (2022). 10.1038/s41539-022-00122-1 PMC9068802 35508471

[5] Youn, C. et al. Genetic Associations with learning over 100 days of practice. *NPJ Sci. Learn.*10.1038/s41539-022-00121-2 (2022). 10.1038/s41539-022-00121-2 PMC9068685 35508486

[6] Schmiedek F, Bauer C, Lovden M, Brose A, Lindenberger U (2010). Cognitive enrichment in old age web-based training programs. Geropsych J. Gerontopsychol. Geriatr. Psychiatry.

[7] Plomin, R. *Blueprint: How DNA Makes Us Who We Are* 1–266 (Mit Press, 2018).

